# Metabolic syndrome is associated with incidence of deep cerebral microbleeds

**DOI:** 10.1371/journal.pone.0194182

**Published:** 2018-03-08

**Authors:** Shingo Mitaki, Hiroyuki Takayoshi, Tomonori Nakagawa, Atsushi Nagai, Hiroaki Oguro, Shuhei Yamaguchi

**Affiliations:** 1 Department of Neurology, Shimane University School of Medicine, Izumo, Japan; 2 Department of Laboratory Medicine, Shimane University School of Medicine, Izumo, Japan; Taipei Veterans General Hospital, TAIWAN

## Abstract

Metabolic syndrome (MetS) has been associated with silent brain lesions; however, there are no data on the relationship between MetS and the incidence of cerebral microbleeds (CMBs) in Asian populations. The aim of this study was to evaluate the longitudinal association between MetS and incidence of CMBs in the Japanese population. We performed a prospective cohort study involving 684 Japanese participants (mean age, 61.7 years) with a mean 6.5 ± 3.4 years follow-up. All participants underwent 1.5 T magnetic resonance imaging, and CMBs were classified by their locations. Logistic regression analyses were performed to examine the relationship of MetS and its components with the incidence of CMBs. MetS was observed in 7.5% of the study population. Forty-nine (7.2%) subjects (36 had new deep or infratentorial CMBs, 13 had new strictly lobar CMBs) developed new CMBs during the follow-up period. In multivariable analysis, MetS was significantly associated with the incidence of deep or infratentorial CMBs (odds ratio, 4.03; 95% confidence interval, 1.72–9.41), and the elevated blood pressure component was most robustly associated with the incidence of deep or infratentorial CMBs (odds ratio, 5.16; 95% confidence interval, 2.02–13.2). Increased body mass index was also associated with incidence of deep or infratentorial CMBs (odds ratio, 2.45; 95% confidence interval, 1.06–5.67). The present study showed that MetS predicts incidence of CMBs in the deep brain regions and high blood pressure is the most important among the MetS components.

## Introduction

Metabolic syndrome (MetS), defined as a complex of modifiable vascular risk factors including central obesity, dyslipidemia, elevated blood pressure, and impaired glucose tolerance, has been reported to predict higher cardiovascular mortality [1]. Previous epidemiological and clinical studies have indicated that MetS is highly correlated to the occurrence [2, 3] and recurrence [4] of stroke. In addition to symptomatic stroke, MetS is also reported to be associated with the prevalence of silent brain lesions, including silent brain infarction and white matter lesions [5].

Cerebral microbleeds (CMBs) detected as small, ovoid, hypointense lesions on T2*-weighted magnetic resonance imaging (MRI), are histologically correlated with focal hemosiderin deposition and considered to represent lipofibrohyalinosis or amyloid angiopathy, depending on their locations [6]. CMBs are generally asymptomatic, but are strong predictive factors of future recurrent hemorrhagic and ischemic stroke [7]. CMBs are also detected in healthy subjects [8], although less frequently than in patients with stroke, and it is notable that the presence of CMBs is a strong independent risk factor for subsequent strokes even in subjects without a history of cerebrovascular disease [8]. Although the clinical implications of CMBs have led to concerns about identifying its risk factors, as far as we are aware, no study has examined the link between MetS and the incidence of CMBs in an Asian population. Thus, the present study aimed to investigate the effects of MetS and its individual components on the incidence of CMBs in Japanese participants.

## Materials and methods

### Study population

**Fig 1:**
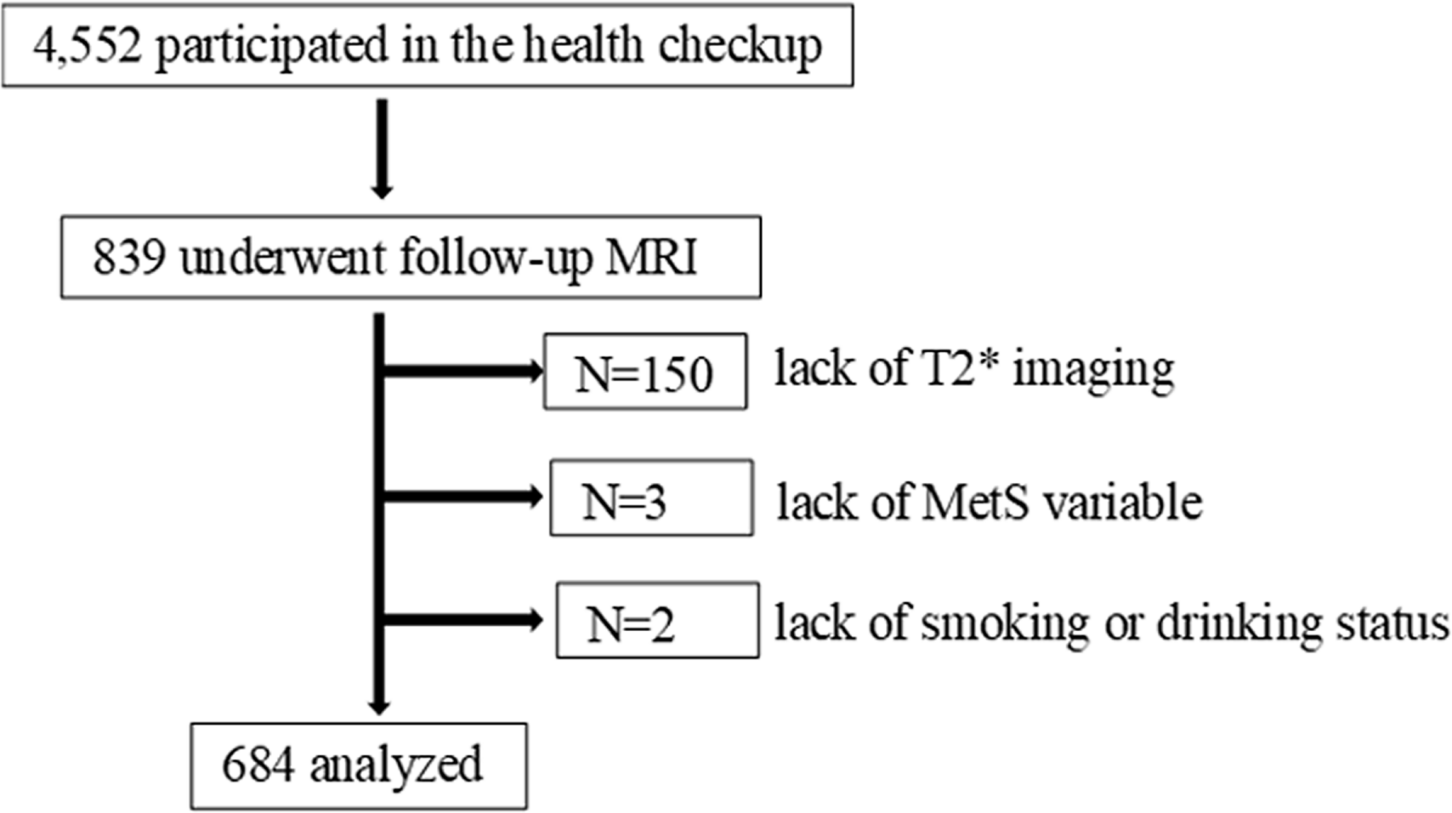
Flow diagram of the study population. MRI: magnetic resonance imaging, MetS: metabolic syndrome.

Our prospective cohort study was approved by the Ethics Committee of the Shimane University School of Medicine in Japan and all participants gave written informed consent in accordance with the Declaration of Helsinki. The procedures followed were in accordance with institutional guidelines. From January 2000 to December 2016, a total of 4,552 Japanese subjects voluntarily participated in the health checkup at the Shimane Institute of Health Science. Medical, neurological, and psychiatric histories were collected from the subjects and neurological examinations by an experienced neurologist, head MRI, and blood tests were also performed. Of the 4,552 subjects, 839 underwent follow-up MRIs. Additionally, 150 subjects with lack of comparable T2* imaging, 3 with missing MetS variables, and 2 with missing smoking or drinking status were excluded. The final analysis included 684 participants (383 men and 301 women) aged 32–87 years (mean 61.7 ± 8.3) with whom we could follow-up for at least half a year after the initial examination (Fig 1). Of 684 participants, 12 had history of stroke and 34 had history of cardiovascular diseases. No patient had dementia.

For subjects who participated three or more times in the brain checkup system, we considered the first visit as the baseline and the last visit as the follow-up.

### Assessment of variables

Body mass index (BMI) was calculated as weight in kilograms divided by height in meters squared. Blood samples were obtained after overnight fasting and serum total cholesterol (TC), high density lipoprotein (HDL-C), low density lipoprotein cholesterol (LDL-C), triglyceride (TG), and fasting plasma glucose levels were measured. Hypertension was defined as systolic/diastolic blood pressure levels ≥ 140/90 mmHg or treatment with antihypertensive drugs. Diabetes mellitus was defined as a fasting blood glucose level ≥ 126 mg/dL, a random blood glucose level ≥ 200 mg/dL, or treatment with oral antidiabetic drugs or insulin. Hyperlipidemia was defined as a serum cholesterol level ≥ 220 mg/dL or treatment with lipid-lowering drugs. Smoking and drinking status were defined as follows: a smoker who at the time smoked at least 1 cigarette per day habitually and a drinker who consumed ≥ 180 mL of alcohol per day, respectively.

### Definition of metabolic syndrome

We defined MetS according to the criteria proposed by the National Cholesterol Education Program Adult Treatment Panel III [9] modified for the Japanese population [10]. Since waist circumference was not measured at the time of data collection and the adequate correlation between waist circumference and BMI were previously demonstrated in the same population [11], we used BMI as a substitute for waist circumference. Based on the previous results [11], central obesity was defined as BMI ≥ 25 kg/m^2^ for men and BMI ≥ 29 kg/m^2^ for women.

### Imaging data

MRIs of the brain were performed on a 1.5 T MRI scanner (Symphony Ultra Gradient; Siemens AG, Erlangen, Germany). The whole brain of each participant was scanned with a slice thickness of 7 mm using gradient- echo T2 * WI (TR: 670 ms, TE: 25 ms). CMBs were identified as 2–10 mm diameter homogenous rounded hypointense lesions on T2 * WI. The locations of the CMBs were classified into three groups: lobar CMBs (cortical gray and subcortical white matter), deep CMBs (deep gray matter: basal ganglia and thalamus and the white matter of the corpus callosum, internal, external, and extreme capsule), and infratentorial (brain stem and cerebellum). The number of CMBs at the baseline MRI was subtracted from the number of those at the follow-up MRI, and if this value was ≥ 1, the individual had an incident CMB. All the MRI findings were read and determined separately by an experienced neurologist and neuroradiologist.

### Statistical analyses

Characteristics of study participants were compared by MetS status with the student *t* test or Fisher’s exact test. We categorized CMBs as follows: “deep or infratentorial CMBs” of participants with one or more CMBs in a deep or infratentorial location regardless of the presence or absence of lobar CMBs, and “strictly lobar CMBs” of participants with one or more CMBs restricted to a lobar location. To generate the odds ratio, univariate logistic regression analysis was conducted at first to assess MetS, MetS components, and other demographic factors for their contribution to the incidence of CMBs. Further, logistic regression models were constructed to determine the association of MetS and MetS components with the incidence of CMB after adjusting for the confounding factors. The statistical analyses were performed using SPSS version 22 (IBM Corp., Armonk, NY). A value of P < 0.05 was considered statistically significant.

## Results

### Baseline characteristics

The baseline characteristics of the 684 study participants are given in Table 1. MetS was present in 7.5% of the study population. Participants with MetS were more likely to be male (96.1% vs 52.8%; P < .0001). Participants with MetS were more likely to be current smokers (23.5% vs 11.7%; P = 0.03) and current drinkers (33.3% vs 17.2%; P = 0.008). Predictably, each diagnostic criterion of MetS was more prevalent in participants with MetS (P < .001). Participants with MetS also had lower baseline levels of TC (200.2 ± 31.2 vs 209.5 ± 30.6 mg/dL; P = 0.04) and LDL-C (109.4 ± 33.1 vs 123.5 ± 28.9 mg/dL; P = 0.04). CMBs were observed in 41 (6.0%) of 684 participants. Of those with CMBs, 38 (92.7%) had deep or infratentorial CMBs and 3 (7.3%) had strictly lobar CMBs. Participants with MetS were more likely to have any CMBs compared to those without MetS (13.7% vs 5.4%; P = 0.03), and they also showed a tendency to have deep or infratentorial CMBs (11.8% vs 5.1%; P = 0.055).

**Table 1:** Characteristics of the 684 study participants at baseline.

Characteristic	with MetS (n = 51)	without MetS (n = 633)	p value
Age (years)	60.6 ± 8.6	61.9 ± 8.3	ns
Male sex (%)	96.1	52.8	< 0.0001
Body Mass Index	27.1 ± 1.7	22.8 ± 2.7	< 0.0001
Hypertension (%)	76.5	38.9	< 0.0001
Systolic Blood Pressure (mmHg)	142.0 ± 12.5	126.5 ± 17.6	< 0.0001
Diastolic Blood Pressure (mmHg)	82.0 ± 8.4	71.9 ± 10.5	< 0.0001
Diabetes Mellitus (%)	27.5	6.8	< 0.0001
Fasting Plasma Glucose (mg/dL)	116.8 ± 25.7	101.8 ± 20.5	< 0.0001
Hyperlipidemia (%)	41.2	44.1	ns
Total Cholesterol (mg/dL)	200.2 ± 31.2	209.5 ± 30.6	0.04
High Density Lipoprotein (mg/dL)	49.4 ± 11.7	62.9 ± 15.4	< 0.0001
Low Density Lipoprotein (mg/dL)	109.6 ± 33.1	124.3 ± 28.4	0.001
Triglyceride (mg/dL)	205.6 ± 132.7	111.6 ± 62.2	< 0.0001
Current Smoker (%)	23.5	11.7	0.03
Current Drinker (%)	33.3	17.2	0.008
Cerebral Microbleeds			
Any (%)	13.7	5.4	0.03
Deep or infratentorial (%)	11.8	5.1	ns
Strictly Lobar (%)	2.0	0.3	ns

Plus–minus value are means ± SD. MetS: metabolic syndrome

### Association of MetS and its components with the incidence of CMBs

The follow-up data showed that 49 participants developed new CMBs during the follow-up period with a mean value of 6.5 ± 3.4 years; 36 had new deep or infratentorial CMBs, 13 had new strictly lobar CMBs. Table 2 provides the odds of having new CMBs for participants with MetS or its components compared to those without. First, we investigated the association between MetS and the incidence of CMBs. In univariate logistic analysis, MetS was associated with the incidence of deep or infratentorial CMBs. After adjusting for confounding factors including follow-up interval, age, sex, current smoking, and current drinking, MetS was still significantly associated with the incidence of deep or infratentorial CMBs. Participants with MetS were 4.03 times more likely to develop new deep or infratentorial CMBs. However, no associations were found between MetS and the incidence of strictly lobar CMBs. Then, we examined the effects of MetS components on the incidence of CMBs. In univariate logistic analysis, increased BMI, elevated blood pressure, and elevated fasting glucose were associated with the incidence of deep or infratentorial CMBs. After all four components were included simultaneously in a logistic regression model with adjustment for age, sex, follow-up interval, current smoking, and current drinking, increased BMI and elevated blood pressure were independent risk factors for the development of deep or infratentorial CMBs (odds ratio (OR) 2.45, 95% confidence interval (CI) 1.06–5.67, OR 5.16, 95% CI 2.02–13.2, respectively). In addition, there was a clear trend toward an increased incidence of deep or infratentorial CMBs with increasing number of MetS components; compared to patients without any MetS components, those having three or more components were almost 14.1 times more likely to develop new deep or infratentorial CMBs.

**Table 2:** Association of MetS and its components with the incidence of CMBs.

	Incidence of any CMBs (n = 49)		Incidence of deep or infratentorial CMBs (n = 36)		Incidence of strictly lobar CMBs (n = 13)	
	OR (95% CI)	p value	OR (95% CI)	p value	OR (95% CI)	p value
MetS						
Univariate	4.31 (2.05–9.06)	< 0.0001	5.69 (2.57–12.6)	<0.0001	1.04 (0.13–8.12)	ns
Multivariable^+^	3.64 (1.62–8.17)	0.002	4.03 (1.72–9.41)	0.001	1.49 (0.17–13.0)	ns
Components						
Univariate						
Increased BMI	3.60 (1.95–6.66)	<0.0001	4.49 (2.25–8.97)	<0.0001	1.51 (0.41–5.59)	ns
Elevated blood pressure	3.96 (2.03–7.74)	<0.0001	6.37 (2.62–15.5)	<0.0001	1.38 (0.46–4.14)	ns
Dyslipidemia	1.16 (0.60–2.24)	ns	1.06 (0.49–2.30)	ns	1.42 (0.43–4.67)	ns
Elevated fasting glucose	1.86 (0.98–3.51)	ns	2.09 (1.02–4.30)	0.04	1.20 (0.33–4.43)	ns
Multivariable^++^						
Increased BMI	2.84 (1.34–6.06)	0.007	2.45 (1.06–5.67)	0.04	2.49 (0.53–11.7)	ns
Elevated blood pressure	3.07 (1.52–6.20)	0.002	5.16 (2.02–13.2)	0.001	1.09 (0.35–3.42)	ns
Dyslipidemia	1.08 (0.38–3.06)	ns	0.52 (0.15–1.77)	ns	7.42 (1.01–54.3)	ns
High density lipoprotein*	0.77 (0.53–1.13)**	ns	0.70 (0.45–1.10)**	ns	0.91 (0.45–1.83)**	ns
Low density lipoprotein*	0.83 (0.63–1.09)**	ns	0.76 (0.57–1.03)**	ns	1.23 (0.61–2.48)**	ns
Triglyceride*	0.72 (0.46–1.14)**	ns	0.88 (0.52–1.47)**	ns	0.39 (0.16–0.98)**	ns
Elevated fasting glucose	1.26 (0.62–2.54)	ns	1.20 (0.53–2.70)	ns	1.44 (0.36–5.74)	ns
No. of Mets components^+^						
0	1.00 (ref)		1.00 (ref)		1.00 (ref)	
1	2.89 (1.03–8.08)	0.04	4.91 (1.07–22.4)	0.04	1.41 (0.33–6.14)	ns
2	4.21 (1.44–12.3)	0.009	7.03 (1.50–32.9)	0.01	1.92 (0.37–10.1)	ns
≥ 3	8.67 (2.81–26.8)	< 0.0001	14.1 (2.93–67.8)	0.001	3.53 (0.51–24.6)	ns

^+^ adjusted for age, sex, follow–up time interval, current smoking, and current drinking.

^++^All four components were included simultaneously in a logistic regression model with adjustment for age, sex, follow–up time interval, current smoking, and current drinking.

CMBs: cerebral microbleeds, MetS: metabolic syndrome, BMI: body mass index, OR: odds ratios, CI: confidence interval, ns: non-significant

*Log-transformed before analysis to reduce skew

** per SD increase

## Discussion

In our prospective cohort study with Japanese participants, we found that the presence of MetS was associated with a 4.03 times increased risk of deep or infratentorial CMBs after adjusting for confounding factors. To the best of our knowledge, this is the first report to show the association of MetS with the incidence of CMBs in an Asian population. Our results would suggest that microstructural brain tissue damage detected in early manifest MetS [12] results in visible brain damage over time. Moreover, when considering the previous findings that MetS conferred additional risk on developing CMBs only when combined with age in Caucasians [13] together with our results, the impact of MetS on CMBs development could vary by ethnicity. However, the impact of MetS on CMBs progression was not above that of its individual components, such as of elevated blood pressure. A few studies have evaluated the association between MetS and silent brain lesions, but the connection is debatable. In one cross-sectional study targeting a Korean population, MetS was reported to be significantly associated with the prevalence of silent brain infarction; however, the impact of elevated blood pressure was superior to that of MetS [14]. On the other hand, a cohort study by Dearborn et al, demonstrated that after adjustment for hypertension, MetS was still an independent risk factor especially for the incidence of larger size of silent lacunar infarctions in a Caucasian population [15]. This discrepancy from our results could be explained by differences in pathological heterogeneity; although deep CMBs and silent lacunar infarction are both responsible for cerebral small vessel diseases, deep CMBs are more homogeneous; to wit, they better and more accurately reflect hypertensive arteriosclerosis. Although it remains debatable whether MetS is a distinct pathologic entity or simply represents an accumulation of risk factors, our results suggest that MetS as a cluster is not superior to the individual components of the syndrome with respect to risk prediction of CMBs.

Nevertheless, detection of one of the modifiable factors for CMB progression in Japanese participants has great significance because of its impact on the risk for future intracranial hemorrhage. Unlike silent brain infarcts, which are predictors of future symptomatic stroke, most frequently ischemic stroke [16], CMBs are reliable precursors of intracranial hemorrhage [8]. Furthermore, there is a clear link between the number of CMBs and the risk of intracranial hemorrhage recurrence [17]. Intracerebral hemorrhages account for approximately 12% of all strokes [18] and their substantial morbidity and mortality exceed that of ischemic stroke [19], and the incidence of intracerebral hemorrhage is 2-fold higher in Asians, and especially in the Japanese [20] than in other ethnicities.

Regarding the individual contributions of the MetS components, elevated blood pressure was most robustly associated with deep or infratentorial CMBs. This finding is in line with data from another population based study [21]. Considering hypertension has been linked to degenerative changes in the arterioles, our results are plausible. Increased BMI was also associated with the incidence of deep or infratentorial CMBs. Whether there is a positive association between incidence of CMBs and BMI or not is controversial. In a longitudinal study that included participants of the AGES-Reykjavik study, the authors found no change in CMB incidence according to basal determinations of BMI [22]. This discrepancy could be explained by differences in ethnicity, age, and follow-up duration; as shown in our study, in middle aged Japanese populations, obesity could be a risk factor for deep CMBs. The effect of ethnic differences on CMBs are also reinforced by the results of a previous study that demonstrated the link between deep CMBs and obesity in Asian populations [23]. The exact mechanism underlying the relationship between BMI and CMBs remains to be elucidated. A previous study suggested that increased metalloproteinase secretion and inflammation associated with obesity may elicit endothelial damage and promote microaneurysm formation, which are pathological substrates capable of explaining the occurrence of CMBs [23].

Unlike elevated blood pressure and increased BMI, other components, such as dyslipidemia and elevated fasting glucose, were not associated with the incidence of CMBs. Previously, in a cross-sectional study, we reported that lower HDL-C levels were associated with a high prevalence of deep CMBs [24]. Although a similar trend was seen in the association between HDL-C levels and incidence of deep or infratentorial CMBs, these association were not significant. Longer follow-up duration with larger samples may be needed to elucidate the biological plausibility of the relationship between HDL-C and incidence of CMBs in deep locations. Moreover, the different functional properties of HDL-C on arteriosclerogenesis or amyloid formation [24] may be responsible for the opposite association of HDL-C with the incidence of CMBs depending on location [22]. As was shown in another cross-sectional [25] and two cohort studies [22, 26], there is no association between elevated fasting glucose and CMBs. These observations may reflect the findings that the association between diabetes mellitus and cerebral small vessel disease is mostly responsible for ‘branch atheromatous disease’ due to microatheroma at the orifice of the penetrating artery, and not for lipohyalinosis.

Our study has several limitations. First, participants in this study were recruited among individuals who participated in the brain checkup; therefore, they may have had different demographic characteristics (e.g., motivation to seek health care and higher socioeconomic status) than participants included in other population-based cohort studies. Thus, our participants may not be representative of the entire Japanese population. Second, we only evaluated MetS at baseline; therefore, we were unable to assess the effect of change in metabolic state on CMBs progression. Third, the possibility of residual confounding by undetermined factors, for example, diet or physical activity, which may have some influence on the metabolic state could not be excluded. Fourth, we did not include detailed information about the subjects’ medications (e.g., antiplatelet agents or antihypertensive therapy) that may have some influence on CMBs. Finally, we only evaluated development of dichotomized CMBs; therefore, we did not evaluate the possible dose–response effect according to the number of CMBs.

In summary, although not beyond the risk associated with its blood pressure components, MetS predicts incidence of deep or infratentorial CMBs. Future cohort studies are needed to validate our results and to facilitate public health prevention or intervention programs to reduce the incidence of CMBs.

## Supporting information

S1 Table Data set used in this study. (XLSX)
